# Adult-onset Still’s disease with hemophagocytic lymphohistiocytosis and minimal change disease

**DOI:** 10.1515/biol-2025-1228

**Published:** 2026-01-01

**Authors:** Dong-hua Guo, Li Wei, Jun-ya Jia

**Affiliations:** Department of Nephrology, Kidney Disease Medical Center, General Hospital, Tianjin Medical University, National Key Clinical Specialty, Tianjin Key Medical Discipline, Tianjin 300052, China

**Keywords:** adult-onset Still’s disease, Epstein-Barr virus, ferritin, hemophagocytic syndrome, minimal change disease

## Abstract

Hemophagocytic lymphohistiocytosis (HLH) is a life-threatening hyperinflammatory syndrome characterized by diverse etiologies and a high risk of misdiagnosis. Autoinflammatory disorders, such as adult-onset Still’s disease (AOSD), are often underrecognized as contributing factors. Minimal change disease (MCD) represents an exceedingly rare renal complication in the context of HLH. This report describes the case of an adult female diagnosed with Epstein–Barr virus (EBV)-associated HLH. Initial therapy with etoposide and dexamethasone yielded a partial response; however, disease relapse occurred, accompanied by the development of significant proteinuria. Renal biopsy confirmed a diagnosis of MCD. Administration of rituximab led to complete remission of the nephrotic syndrome, although HLH activity persisted. A subsequent skin biopsy revealed perivascular infiltration composed predominantly of lymphocytes and neutrophils. In combination with clinical features such as high-grade fever and rash, these findings supported a diagnosis of AOSD. Adjustment of the treatment regimen to include corticosteroids and methotrexate resulted in complete remission of HLH. This case underscores the importance of re-evaluating the underlying cause in patients with HLH who demonstrate an inadequate therapeutic response. MCD may represent a renal manifestation of immune dysregulation associated with HLH. A multidisciplinary approach is essential for improving diagnostic precision and optimizing treatment in complex presentations of HLH.

## 1 Introduction

Hemophagocytic lymphohistiocytosis (HLH) is a life-threatening syndrome characterized by dysregulated and excessive systemic inflammation, often with multifactorial etiologies. Approximately 50 % of patients with HLH develop the condition secondary to infections, malignancies, or autoimmune disorders [1]. Among these, adult-onset Still’s disease (AOSD), a rare autoinflammatory disorder, presents a diagnostic challenge when occurring in conjunction with HLH, as it is frequently misdiagnosed as infection or hematologic malignancy. The rate of missed diagnosis has been reported to be as high as 30 % [2].

HLH has been shown to involve multiple organ systems; however, the development of minimal change disease (MCD) remains rare, with only sporadic cases documented globally. The underlying pathogenesis is thought to involve T cell–mediated podocyte injury [3]. When HLH, MCD, and AOSD coexist, their overlapping etiologies and potentially conflicting treatment requirements substantially complicate clinical management. Infections such as Epstein–Barr virus (EBV) may serve as shared triggers for both HLH and MCD, while cytokine-driven hyperinflammation in AOSD may further exacerbate renal injury.

At present, standardized diagnostic and therapeutic protocols for such complex clinical scenarios are lacking, and the role of multidisciplinary care in their management has not been well defined. This report presents a diagnostically and therapeutically challenging case that evolved from EBV-associated HLH with concurrent MCD to a final diagnosis of AOSD. A 3-year follow-up underscores the importance of ongoing etiological reassessment and a stepwise treatment approach, contributing to a broader understanding of the immune regulatory mechanisms underlying HLH–MCD–AOSD comorbidity.

## 2 Case description

A 24-year-old female patient was admitted to the intensive care unit on May 8, 2021, with a history of intermittent rash for more than one month and intermittent fever for 20 days. One month prior to admission, she developed a rash on the extremities accompanied by pruritus, which recurred following treatment. Twenty days prior to admission, she developed fever with a maximum temperature of approximately 38 °C and urticarial lesions on the extremities. She was diagnosed with acute urticaria at an external hospital. During the two weeks preceding admission, her fever worsened, reaching 39.2 °C, and was associated with throat dryness and sore throat. Urinalysis revealed BLD 2+ and PRO 2+. Imaging studies demonstrated small pulmonary nodules and mild splenomegaly. The patient declined hospitalization at that time. Subsequently, her condition deteriorated, with the onset of blurred vision and hemodynamic instability consistent with shock, leading to admission to the intensive care unit with a working diagnosis of fever of unknown origin and shock.

**Informed consent:** Informed consent has been obtained from all individuals included in this study.

**Ethical approval:** The research related to human use has been complied with all the relevant national regulations, institutional policies and in accordance with the tenets of the Helsinki Declaration, and has been approved by the Ethics Committee of the General Hospital of Tianjin Medical University (IRB2025-YX-514-01).

### 2.1 Past medical history

The patient reported recurrent oral ulcerations. There was no history of chronic illness, including hypertension, diabetes mellitus, or coronary artery disease. She denied tobacco use. The patient was unmarried and nulliparous. No relevant family history was identified.

### 2.2 Physical examination on admission

Vital signs were as follows: body temperature 37.9 °C, heart rate 111 beats/min, respiratory rate 20 breaths/min, and blood pressure 112/67 mmHg. No jaundice was noted in the skin or mucous membranes. Oral examination revealed multiple white patches on the right buccal mucosa, with partial confluence. Palpation identified enlarged left posterior cervical lymph nodes measuring approximately 1 × 1 cm, with local tenderness, firm consistency, and mildly reduced mobility. Additionally, mobile, non-tender left axillary lymph nodes measuring approximately 0.5 × 0.5 cm were noted, with acceptable consistency. The neck was supple. Cardiopulmonary and abdominal examinations were unremarkable. No pitting edema was present in the lower extremities.

## 3 Initial laboratory findings (Emergency Department, day before intensive care unit admission)

Initial laboratory evaluation demonstrated leukocytosis with a white blood cell count (WBC) of 13.89 × 10^9^/L and neutrophil predominance of 92.0 %. Hemoglobin (Hb) was 140 g/L and platelet count (PLT) was 195 × 10^9^/L. Inflammatory markers were elevated, with procalcitonin (PCT) 18.36 ng/mL and C-reactive protein (CRP) 112.71 mg/L. Blood biochemistry revealed hypoalbuminemia (albumin, ALB 30 g/L), elevated transaminases (alanine aminotransferase, ALT 129 U/L; aspartate aminotransferase, AST 327 U/L), increased lactate dehydrogenase (LDH 848 IU/L), and impaired renal function (creatinine, Cr 204 μmol/L). Electrolyte abnormalities included hypokalemia (K 3.0 mmol/L) and hypocalcemia (Ca 1.80 mmol/L). Coagulation testing showed a prolonged prothrombin time (PT) of 15.6 s, fibrinogen 4.31 g/L, and markedly elevated D-dimer (>10,000 ng/mL, fibrinogen equivalent units, FEU).

## 4 Laboratory findings and clinical course following admission to the Department of Critical Care Medicine

On admission, laboratory evaluation revealed leukocytosis with a WBC count of 10.09 × 10^9^/L and neutrophil predominance of 93.3 %. Hb was 108 g/L. Biochemical analysis showed elevated urea (9.1 mmol/L), increased Cr (129 μmol/L), hypoalbuminemia (ALB 26 g/L), and elevated LDH (1134 U/L). Inflammatory markers remained elevated, with PCT 13.13 ng/mL and CRP 16.6 mg/L.

Autoimmune evaluation demonstrated negative rheumatoid-related antibodies, anti-neutrophil cytoplasmic antibody (ANCA), and anti-glomerular basement membrane (GBM) antibodies were negative. Immunoglobulin G (IgG) was decreased at 719.00 mg/dL, and complement component C3 was reduced at 63.30 mg/dL. Urinalysis revealed hematuria (BLD ±) and proteinuria (PRO 2+), with 24-h urinary protein excretion of 552.0 mg. Ferritin was markedly elevated (>2000.00 ng/mL).

Serological testing indicated past EBV exposure, with positive anti-EBV nuclear antigen and viral capsid antigen (VCA) IgG antibodies. Chest radiography demonstrated increased pulmonary markings bilaterally.

During hospitalization, despite supportive measures and anti-infective therapy, the patient continued to experience intermittent fever. The presence of splenomegaly and markedly elevated ferritin prompted further evaluation. Following multidisciplinary consultation, hematologic disease was strongly suspected, with HLH not excluded. On May 18, 2021, the patient was transferred to the Department of Hematology for further assessment.

## 5 Laboratory findings and clinical course following admission to the Hematology Department

On admission to the Hematology Department, Hb was 104 g/L, WBC 5.27 × 10^9^/L, neutrophils 51.8 %, lymphocytes 24.9 %, monocytes 11.4 %, eosinophils 10.6 %, and reticulocyte percentage 4.34 %. Anti-EBV nuclear antigen and VCA IgG antibodies remained positive. Renal function had improved, with urea 2.8 mmol/L and Cr 68 μmol/L. Serum protein analysis showed total protein 78 g/L, globulin 38 g/L, and persistently low ALB. LDH was 370.8 U/L, triglycerides were elevated at 3.67 mmol/L, and ferritin remained >2000 ng/mL; dilutional testing yielded a concentration of 5,156.66 ng/mL.

Ultrasound of superficial lymph nodes revealed multiple enlarged nodes at cervical levels I–V bilaterally (some with cortical thickening), as well as in the axillary and inguinal regions. Soluble CD25 (sCD25) was markedly elevated at 14,493 pg/mL, and natural killer cell (NK) activity was decreased at 17.48 %. EBV-infected lymphocytes (CD3^−^CD19^+^) were detected at 2.5 × 10^3^.

Positron emission tomography–computed tomography (PET-CT) demonstrated: (1) multiple metabolically active lymph nodes of varying sizes throughout the body, raising suspicion for lymphoma; and (2) abnormally elevated splenic metabolism, suggestive of possible neoplastic infiltration. Bone marrow aspiration (iliac crest) showed increased myeloid and megakaryocytic lineages with erythroid hyperplasia. Bone biopsy revealed scattered lymphocytes and plasma cells, with no morphologic abnormalities in megakaryocytes. Lymph node biopsy demonstrated predominantly fibrovascular adipose tissue with a minor lymphoid component. Collectively, these findings were not consistent with a definitive diagnosis of lymphoma.

The working diagnosis established by the Hematology Department included: hemophagocytic syndrome, possible lymphoma, EBV infection, splenomegaly, fatty liver, minimal pericardial effusion, and minimal pelvic effusion.

Treatment during this admission included anti-infective therapy with sodium phosphonoformate, piperacillin–tazobactam, and linezolid. Immunomodulatory therapy consisted of intravenous immunoglobulin (IVIG), along with supportive measures including hepatoprotective agents, urinary alkalization, and gastric acid suppression. In accordance with the 2004 HLH guidelines, for EBV-associated HLH without overt malignancy, a 4-week course of rituximab (375 mg/m^2^ weekly, alone or with dexamethasone) may be used to rapidly eliminate EBV reservoirs and reduce subsequent etoposide exposure. The patient received rituximab on June 4, June 15, June 25, and July 6, 2021, at doses of 100 mg each, for a cumulative total of 400 mg.

## 6 Laboratory follow-up after five months

At five-month follow-up, laboratory testing demonstrated persistently elevated ferritin (2,285.67 ng/mL) and sCD25 (7,398 pg/mL), with slightly improved NK cell activity (22.93 %). EBV-infected lymphocyte subsets revealed CD3^+^CD8^+^ cells at 1.8 × 10^1^. Based on the clinical and laboratory findings, the HLH-1994 treatment protocol was initiated on October 28, 2021. Therapy included etoposide and dexamethasone. Etoposide was administered at 250 mg twice weekly during the first week, followed by weekly administration from weeks 2 through 10.

## 7 Follow-up after eight months

At eight months, follow-up testing demonstrated a reduction in sCD25 to 3,086 pg/mL, with NK cell activity at 18.81 %. EBV-related lymphocyte subset analysis showed undetectable CD3^+^CD4^+^ and CD3^−^CD19^+^ cells; CD3^+^CD8^+^ cells were present at 6.3 × 10^1^, while CD56^+^ cells were undetectable. Oral cyclosporine (50 mg every 12 h) was added to the treatment regimen.

## 8 Follow-up after nine months

Nine months later, the patient developed new-onset periorbital edema with diurnal variation, worsening in the evening. Clinical evaluation revealed symmetrical, pitting edema. Urinalysis showed albuminuria (4^+^) and trace hematuria (±). Biochemical testing revealed hypertriglyceridemia (2.92 mmol/L), elevated total cholesterol (9.87 mmol/L), hypoalbuminemia (18 g/L), ferritin 218.20 ng/mL, and markedly increased 24-h urinary protein excretion (16,676.0 mg), consistent with nephrotic syndrome.

The patient was transferred to the Department of Nephrology for further evaluation. Immune monitoring revealed sCD25 3,817 pg/mL and decreased NK cell activity (12.66 %). EBV-infected lymphocyte analysis demonstrated CD3^−^CD19^+^ cells at 2.2 × 10^3^, with no detectable CD3^+^CD4^+^, CD3^+^CD8^+^, or CD56^+^ cells.

**Figure 1:**
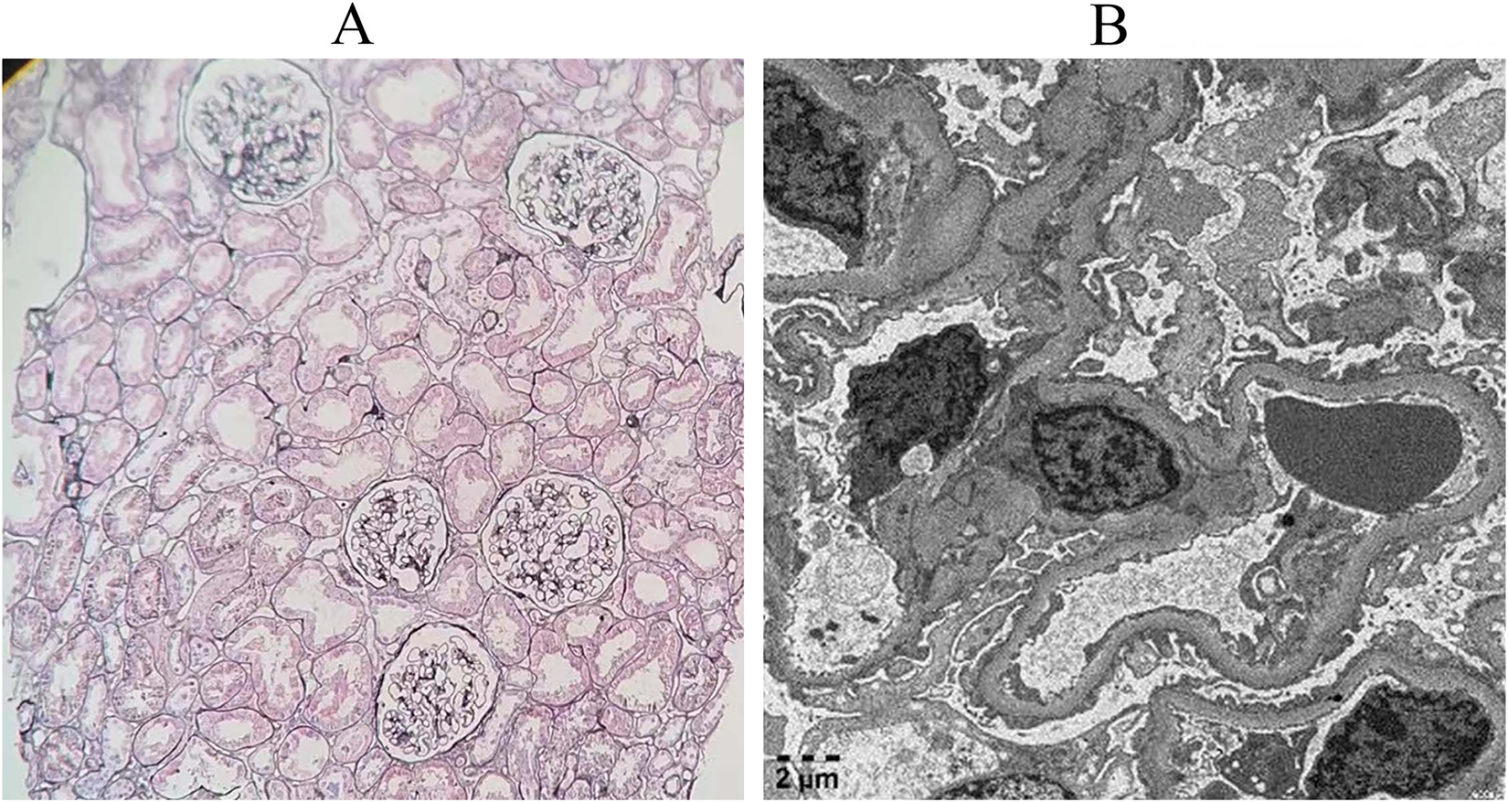
Renal biopsy findings. (A) Light microscopy (×40) with periodic acid–silver methenamine staining revealed ischemic sclerosis in one of 22 glomeruli. The remaining glomeruli demonstrated mild, focal, and segmental mesangial cell and matrix proliferation, with diffuse vacuolar changes of the capillary basement membranes. (B) Electron microscopy (×5,000) showed diffuse effacement of epithelial foot processes. The findings were consistent with minimal change disease, although focal segmental glomerulosclerosis could not be completely excluded.

Renal biopsy (Figure 1A) revealed histopathological features consistent with: (1) MCD and (2) acute tubular injury.

**Light microscopy:** 22 glomeruli were identified, one with ischemic sclerosis; the remaining glomeruli showed mild, focal, and segmental mesangial proliferation with diffuse vacuolar changes of the capillary basement membranes.

**Immunofluorescence:** four glomeruli were available. IgM displayed faint, segmental, finely granular deposits along capillary loops and focal mesangial areas. IgA, IgG, C3, C1q, C4, FRA, and albumin were negative. No specific staining was seen for κ or λ light chains. PLA2R was negative. IgG subclass analysis (IgG1–IgG4) was negative. HBsAg was positive; HBcAg was negative.

**Electron microscopy:** diffuse effacement of epithelial foot processes was observed. The findings were consistent with MCD, although focal segmental glomerulosclerosis could not be completely excluded.

The treatment strategy was adjusted to address both MCD and suspected EBV-related immune dysregulation involving plasma cells. Rituximab was reinitiated at 200 mg weekly for four doses beginning February 28, 2022, supplemented with IVIG. On March 10, 2022, electron microscopy confirmed MCD (Figure 1B). By May 9, 2022, 24-h urinary protein had decreased to 0.03 g, indicating complete remission of nephrotic syndrome.

## 9 Follow-up after one year and five months

Splenomegaly persisted Laboratory evaluation demonstrated ferritin 5,643 ng/mL and sCD25 15,359 pg/mL, with NK cell activity 24.48 %. No abnormalities were detected in EBV-infected lymphocyte subsets. Due to relapsing inflammatory activity, a second course of etoposide (250 mg twice weekly for two weeks) and dexamethasone (10 mg once daily for two weeks) was initiated.

## 10 Follow-up after one year and ten months

The patient developed influenza A infection, confirmed by positive influenza A RNA. EBV-DNA was undetectable, and EBV-infected lymphocyte subset testing showed CD3^+^CD4^+^ cells at 1.1 × 10^1^. Serum ferritin was 944.53 ng/mL. The patient received antiviral therapy and symptomatic treatment.

## 11 Follow-up after two years and six months

The patient was readmitted to the Department of Hematology with fever and rash. *Mycoplasma pneumoniae* DNA was detected, and serum ferritin was elevated at 1,487.31 ng/mL. A diagnosis of *M. pneumoniae* infection was established, and azithromycin was administered.

**Figure 2:**
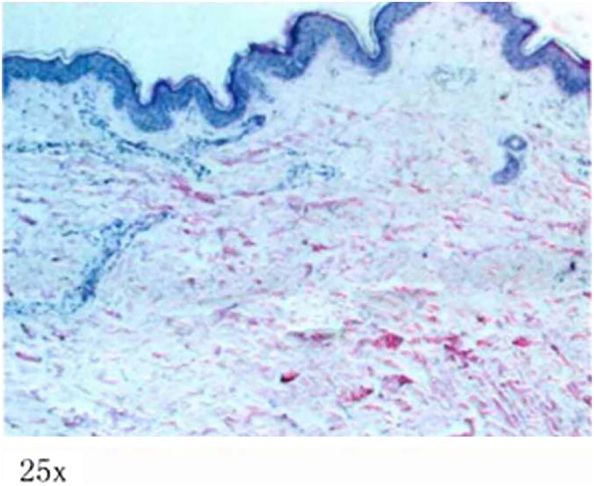
Skin biopsy findings. Hematoxylin and eosin staining (×25) of biopsied erythematous lesions demonstrated inflammation predominantly within the superficial-to-mid dermis, arranged diffusely and around vessels, without involvement of the basal epidermis. Dense infiltrates of mature neutrophils with associated nuclear debris (leukocytoclasia) and scattered lymphocytes were observed, without conspicuous eosinophils, distinguishing the findings from drug reactions or insect-bite hypersensitivity. The vascular endothelium exhibited mild swelling without fibrinoid necrosis or frank leukocytoclastic vasculitis. The epidermis remained intact, lacking spongiform pustules or interface dermatitis, thereby excluding psoriasis and lupus erythematosus.

A skin biopsy of multiple erythematous rashes on the face, neck, and upper limbs suggested findings not excluding AOSD (Figure 2). Concurrent superficial lymph node biopsy demonstrated lymphoid hyperplasia with mild eosinophilic infiltration and active T-zone proliferation. Immunohistochemistry revealed CD20^+^ B cells, CD3^+^ T cells, an organized follicular dendritic cell network (CD21^+^), high Ki-67 expression in germinal centers, and scattered CD68 positivity.

The working diagnoses included: (1) hemophagocytic syndrome, (2) suspected AOSD, (3) possible lymphoma, (4) *M. pneumoniae* infection, (5) nephrotic syndrome, and (6) MCD. Treatment included methylprednisolone 40 mg daily, followed by oral prednisone acetate 25 mg twice daily at discharge, with referral to the Department of Rheumatology and Immunology.

## 12 Course in the Department of Rheumatology and Immunology

In the Rheumatology and Immunology Department, the patient was diagnosed with AOSD. Treatment with corticosteroids and methotrexate resulted in marked reduction of serum ferritin and resolution of lymphadenopathy, indicating clinical improvement. Following dose tapering of corticosteroids and methotrexate, serum ferritin levels remained stable. The patient’s condition has remained stable with ongoing outpatient monitoring. Please refer to Table 1 for details of Clinical course.

**Table 1:** Clinical course of the patient.

Time points	Brief description of the condition
2021-05	24 years old, female. Due to high fever of 39 °C, rash and shock, she was admitted to the ICU. The inflammatory indicators were extremely high, ferritin >5,000 ng/mL, sCD25 ↑, NK activity ↓, positive for EBV past infection markers → suspected HLH, transferred to the Hematology Department
2021-06	Rituximab 100 mg * 4: To control EBV-B cells
2021-10	HLH-1994 protocol: Etoposide + dexamethasone → partial remission
2022-02	Sudden onset of nephrotic syndrome, renal biopsy: MCD → re-administration of rituximab 200 mg * 4 weeks → urine protein turned negative rapidly, but the HLH indicators remained high
2022-10	Ferritin levels rose again to 5,600 ng/mL. The second round of treatment was etoposide combined with dexamethasone
2023-03	Repeated high fever + typical rash → skin/lymph node biopsy: Suspected AOSD → revised diagnosis: AOSD secondary to HLH, combined with EBV, triggered MCD
2023-04	Hormone + methotrexate → iron protein rapidly returned to normal, lymph nodes shrank, and the condition has remained stable up to now (follow-up for 3 years)

ICU, intensive care unit; HLH, hemophagocytic lymphohistiocytosis; MCD, minimal change disease; AOSD, adult-onset Still’s disease.

## 13 Discussion

HLH, also referred to as hemophagocytic syndrome, is a hyperinflammatory disorder caused by inherited or acquired immune dysregulation. It is characterized by pathological activation and proliferation of lymphocytes, monocytes, and macrophages, with subsequent secretion of large quantities of proinflammatory cytokines. Clinically, HLH presents with persistent fever, cytopenia, hepatosplenomegaly, and histological evidence of hemophagocytosis in the bone marrow, liver, spleen, or lymph nodes. HLH is broadly classified into primary and secondary forms.

Primary HLH is associated with pathogenic gene variants that impair cytolytic lymphocyte function or inflammatory regulation and is typically inherited in an autosomal recessive or X-linked pattern. Secondary HLH is precipitated by external triggers such as malignancy, infection, autoimmune or autoinflammatory disease, medications, pregnancy, or transplantation, and is not usually linked to HLH-associated gene mutations or a relevant family history.

The underlying mechanism of HLH involves impaired cytotoxic activity of NK cells and cytotoxic T lymphocytes (CTLs), leading to the failure to eliminate activated macrophages. This results in excessive cytokine production, particularly interferon-gamma (IFN- γ), which drives widespread immune cell activation and tissue infiltration. The patient in this case exhibited hallmark clinical and laboratory features of HLH, including fever, splenomegaly, elevated serum ferritin and sCD25 concentrations, and decreased NK cell activity.

In adults, HLH is most often secondary in nature and commonly associated with hematologic malignancies, rheumatic immune diseases, infections, transplantation, medications, pregnancy, or other identifiable triggers. In the present case, PET-CT initially raised suspicion for lymphoma; however, repeated bone marrow and lymph node biopsies did not confirm this diagnosis. Comprehensive immunologic testing, including rheumatoid factor (RF), a complete immunological panel, ANCA, anti-GBM antibodies, and other relevant markers, did not support an autoimmune or rheumatic etiology. The patient also had no history of transplantation, exposure to specific medications, or pregnancy.

Serological testing confirmed EBV exposure, with CD3^−^CD19^+^ EBV-infected B lymphocytes detected at 2.5 × 10^3^ cells, consistent with active or latent infection. EBV is a well-documented primary trigger of HLH and may also act as a cofactor that exacerbates HLH associated with malignancies or autoimmune disorders. Whether occurring independently or in combination with other conditions, EBV plays a central role in immune dysregulation [4]. Quantitative EBV-DNA testing in peripheral blood mononuclear cells and plasma can help determine etiology and guide therapeutic strategies in HLH. In this case, HLH was initially attributed to EBV-driven immune activation in the absence of other identifiable triggers. Targeted therapy with rituximab was employed to eliminate EBV-infected B cells. As the disease progressed, EBV was observed to have shifted tropism to T cells, leading to initiation of the HLH-1994 treatment protocol, which includes etoposide and dexamethasone.

Approximately 10 months after the initial HLH diagnosis, the patient developed nephrotic syndrome. Renal biopsy confirmed MCD, and concurrent laboratory testing indicated ongoing EBV infection. A central diagnostic consideration was whether MCD developed as a secondary consequence of EBV infection or as a manifestation of HLH-associated immune dysregulation. Viral infections, including EBV, are well recognized as potential triggers of MCD by inducing immune responses that impair podocyte function. EBV in particular has been implicated in various forms of glomerular injury.

Several studies have suggested a potential mechanistic association between HLH and MCD independent of active viral infection. Thaunat et al. retrospectively analyzed 11 patients with HLH complicated by nephrotic syndrome and identified MCD in 4 cases on renal biopsy [3]. None of these patients demonstrated evidence of EBV infection. The reported underlying etiologies of HLH included lymphoma (2 cases), AOSD (1 case), and primary HLH (1 case). In all four patients, HLH was clinically active at the onset of nephrotic syndrome, suggesting that HLH itself may contribute to podocyte injury.

Racial differences in patterns of glomerular injury among patients with HLH have also been observed, with collapsing glomerulopathy reported more frequently in African patients, while MCD is more often described in Caucasian patients [3]. These findings raise the possibility of genetic predisposition influencing renal outcomes in HLH. In the present case, the absence of genetic testing represents a limitation, as such data could have provided additional insight into host susceptibility.

Given the coexistence of EBV-infected B cells at the time of MCD diagnosis, rituximab therapy was continued. A cumulative dose of 800 mg was administered, resulting in complete remission of proteinuria. It is important to note that MCD may not arise directly from HLH itself but rather from the cytokine storm and unregulated T-cell activation associated with HLH.

Despite two courses of rituximab and treatment according to the HLH-1994 protocol, the patient demonstrated only limited clinical improvement. Although fever temporarily subsided and sCD25 and ferritin levels decreased significantly, NK cell activity did not recover. Intermittent fever and recurrent rash persisted, and skin biopsy revealed histopathological features consistent with cutaneous manifestations of AOSD.

AOSD is a rare, multifactorial systemic inflammatory disorder. Reported incidence rates range from 0.16 to 0.40 per 100,000, with prevalence estimated at 1–34 per 1,000,000 [5]. The condition most commonly affects young adults, with two peaks of onset typically reported between 15–25 years and 36–46 years of age [5], 6]. AOSD may be complicated by severe systemic manifestations, including HLH, acute respiratory distress syndrome, diffuse alveolar hemorrhage, disseminated intravascular coagulation, and fulminant hepatitis [7], 8].

The pathogenesis of HLH secondary to AOSD is thought to involve excessive activation of the reticuloendothelial and mononuclear phagocyte systems, frequently triggered by heightened AOSD activity or intercurrent infections. This activation results in macrophage hyperactivation and release of proinflammatory cytokines, including interleukin (IL)-6, IL-1, IL-18, and tumor necrosis factor (TNF)-α, culminating in cytokine storm and multi-organ damage [9]. Furthermore, genetic variants affecting cytotoxic lymphocyte function, such as mutations in *PRF1*, *UNC13D*, and *IRF5*, have been implicated in HLH pathogenesis, particularly in cases lacking identifiable external triggers [10].

The diagnosis of AOSD complicated by HLH requires that diagnostic criteria for both conditions be concurrently fulfilled. AOSD remains a diagnosis of exclusion, with the Yamaguchi criteria most commonly applied [11]. In the present case, the patient met two major criteria – spiking fever and typical rash – and four minor criteria, including sore throat, lymphadenopathy with splenomegaly, abnormal liver function, and negative rheumatoid factor and antinuclear antibody (ANA) tests.

Malignancy, alternative autoimmune diseases, and significant infections were excluded during more than three years of evaluation, supporting the diagnosis of AOSD.

In a retrospective analysis of 447 patients with AOSD, Wang et al. identified several independent risk factors for macrophage activation syndrome (MAS), including platelet count <100 × 10^9^/L, aspartate aminotransferase (AST) >120 U/L, triglycerides >3 mmol/L, ferritin >1,500 ng/mL, and bone marrow hemophagocytosis [2]. Similarly, Bae et al. examined 109 patients with AOSD and concluded that thrombocytopenia, anemia, and hepatomegaly may serve as predictive factors for MAS. The patient described here exhibited anemia, hepatomegaly, persistently elevated ferritin, and elevated AST at disease onset, classifying her as high risk for MAS [12].

The most common cutaneous manifestation of AOSD is a transient salmon-pink or erythematous maculopapular rash. This eruption typically appears during febrile episodes, most often on the proximal extremities and trunk, with less frequent involvement of the face or distal extremities [13].

Histopathologically, typical transient AOSD lesions exhibit preserved epidermis with mild perivascular inflammatory infiltrates in the superficial dermis, composed primarily of lymphocytes and neutrophils [13]. Persistent papules and plaques may demonstrate necrotic keratinocytes in the upper epidermis and dense neutrophilic infiltrates in the papillary dermis [14]. Urticarial variants are characterized by perivascular interstitial neutrophilic infiltrates, consisting of mature CD15^+^ neutrophils distributed between dermal collagen bundles. Although leukocytoclasia may be observed, the absence of vascular wall damage distinguishes these findings from true vasculitis [15].

While such histopathological features may support the diagnosis, they are not pathognomonic and are not considered definitive diagnostic criteria for AOSD. In the present case, rash was observed at disease onset, but no biopsy was performed at that time. As AOSD is a diagnosis of exclusion, the absence of histological confirmation does not preclude diagnosis. The cumulative diagnostic and therapeutic findings observed during the subsequent clinical course were instrumental in establishing a definitive diagnosis of AOSD.

The differential diagnosis of AOSD includes sepsis, infections, malignancies, familial hemophagocytic lymphohistiocytosis (FHL), and other autoimmune diseases. In its early stages, HLH may clinically mimic sepsis, often resulting in misdiagnosis. However, features such as hyperferritinemia, splenomegaly, and elevated leukocyte antigen-DR expression may help distinguish HLH from sepsis [15]. In this patient, normal WBC count and PCT levels, together with markedly elevated ferritin and splenomegaly, made a primary diagnosis of sepsis less likely.

Infections, particularly viral infections such as EBV, are recognized triggers of both HLH and AOSD. Although EBV infection was identified early in the disease course, the patient’s condition did not resolve following antiviral therapy and standard HLH-directed treatment. Thus, while EBV likely contributed to disease initiation, it was insufficient to explain the persistent and evolving clinical phenotype.

Malignancy-associated HLH, most frequently secondary to lymphoma, typically progresses rapidly and carries a poor prognosis. This possibility was considered early but was excluded based on negative findings from bone marrow aspiration, lymph node histology, and imaging studies.

FHL is a genetic disorder, usually associated with family history, rapid clinical deterioration, and poor prognosis, and is typically differentiated by NK cell function testing and genetic analysis. These features were not present in the current case.

Other autoimmune diseases were also considered. The patient did not exhibit malar or discoid rash, photosensitivity, oral ulcers, arthritis, or sicca symptoms. A comprehensive autoimmune antibody panel was entirely negative, and hypergammaglobulinemia was absent. Furthermore, renal biopsy did not demonstrate a “full-house” pattern of immune-complex deposition, effectively excluding systemic lupus erythematosus, Sjögren’s syndrome, and related autoimmune conditions.

The absence of a family history, combined with the patient’s sustained therapeutic response and clinical stability, makes FHL unlikely. Considering the medical history, laboratory findings, and response to immunosuppressive therapy, the final diagnosis was AOSD complicated by secondary HLH.

Following initiation of corticosteroids and methotrexate under the care of the rheumatology and immunology team, the patient achieved clinical remission, with normalization of body temperature and serum ferritin, resolution of lymphadenopathy, and disappearance of the cutaneous rash. The patient continues to be monitored in the outpatient setting, with stable disease control.

In this case, the patient initially presented with HLH, presumed to be secondary to EBV infection. Despite antiviral therapy and standard HLH-directed treatment, the condition remained refractory. During the disease course, the patient developed nephrotic syndrome, with renal biopsy confirming MCD. Rituximab therapy induced remission of nephrotic syndrome; however, HLH activity persisted. A subsequent skin biopsy revealed findings compatible with AOSD, and the patient experienced marked clinical improvement following standard AOSD-directed therapy.

This case illustrates the diagnostic complexity of HLH and emphasizes the importance of continuously reassessing underlying etiologies in patients with inadequate response to standard treatment. Although MCD may not arise directly from HLH, it can result from immune dysregulation driven by cytokine storm and uncontrolled T-cell activation. Given that HLH encompasses syndromes with diverse etiologies, accurate identification of the inciting factor is essential for guiding targeted therapy.

In this patient, peripheral blood EBV-DNA was repeatedly detected at low levels (<1,000 copies/mL), while plasma EBV-DNA remained negative, suggesting the virus did not enter a high-level lytic phase and arguing against a direct causal link to active EBV-HLH. EBER-ISH negativity in lymph node and skin biopsies further excluded EBV-driven lymphoproliferative lesions. Taken together – viral load, histology, immunologic findings, and therapeutic response – EBV appeared to act more as a marker of immune dysregulation and a co-amplifier, rather than as the sole causal trigger. In this case, EBV initiated the process, HLH sustained the cytokine storm, and AOSD perpetuated inflammation, collectively driving systemic disease and podocyte injury (MCD). Long-term remission thus requires simultaneous disruption of EBV activity, HLH-related hypercytokinemia, and the self-sustaining inflammatory loop of AOSD.

A critical appraisal of this case identifies several limitations:

1) Attribution to EBV without quantitative confirmation: EBV-IgG/VCA positivity indicates past infection, but quantitative EBV-DNA in peripheral blood was not consistently measured, and high viral loads or tissue EBER positivity were not demonstrated. Over-reliance on EBV serology as a causal explanation for HLH is a common diagnostic pitfall.
2) Lack of genetic evaluation: Persistently reduced NK-cell activity was not followed by targeted HLH-gene analysis (e.g., *PRF1*, *UNC13D*, *STX11*, *STXBP2*, *RAB27A*, *LYST*, *AP3B1*, *MAGT1*) or whole-exome sequencing. Functional assays such as flow-cytometric assessment of perforin, granzyme B, and SAP/XIAP protein expression were also not performed, despite their utility in rapidly distinguishing primary HLH.
3) Limited skin-biopsy characterization: The report noting findings “not excluding AOSD” lacked quantitative assessment of neutrophilic infiltrates and markers such as MPO and CD68, reducing diagnostic specificity.
4) Restricted immunologic profiling in AOSD: The diagnosis of AOSD relied solely on Yamaguchi criteria. Biomarkers such as IL-18, IL-1β, IL-6, and MRP8/14 were not measured. Notably, IL-18 concentrations >40,000 pg/mL have been reported to carry high specificity for AOSD complicated by MAS.
